# Gathering patients and rheumatologists' perceptions to improve outcomes in idiopathic inflammatory myopathies

**DOI:** 10.1016/j.clinsp.2022.100031

**Published:** 2022-04-11

**Authors:** Isabela M. Bertoglio, Glaucia F. Abrahao, Fernando H.C. de Souza, Renata Miossi, Paloma C. de Moraes, Samuel K. Shinjo, Eloisa Bonfá, Michelle R. Ugolini Lopes

**Affiliations:** Rheumatology Division, Hospital das Clínicas, Faculdade de Medicina, Universidade de São Paulo (HCFMUSP), São Paulo, SP, Brazil

**Keywords:** Idiopathic inflammatory myopathies, Patient reported outcome measure, Treat to target, Outcome concerns

## Abstract

**Objective:**

Therapeutic targets in Idiopathic Inflammatory Myopathies (IIM) are based on the opinions of physicians/specialists, which may not reflect the main concerns of patients. The authors, therefore, assessed the outcome concerns of patients with IIM and compared them with the concerns of rheumatologists in order to develop an IIM outcome standard set.

**Methods:**

Ninety-three IIM patients, 51 rheumatologists, and one physiotherapist were invited to participate. An open questionnaire was initially applied. The top 10 answers were selected and applied in a multiple-choice questionnaire, inquiring about the top 3 major concerns. Answers were compared, and the agreement rate was calculated. Concerns were gathered in an IIM outcome standard set with validated measures.

**Results:**

The top three outcome concerns raised by patients were medication side effects/muscle weakness/prevention functionality loss. The top three concerns among rheumatologists were to prevent loss of functionality/to ensure the quality of life/to achieve disease remission. Other's outcomes concerns only pointed out by patients were muscle pain/diffuse pain/skin lesions/fatigue. The agreement rate between both groups was 41%. Assessment of these parameters guided the development of an IIM standard set which included Myositis Disease Activity Assessment Visual Analogue Scale/Manual Muscle Testing/fatigue and pain Global Visual Analogue Scale/Health Assessment Questionnaire/level of physical activity.

**Conclusion:**

The authors propose a novel standard set to be pursued in IIM routine follow-up, which includes not only the main patients/rheumatologist outcome concerns but also additional important outcomes only indicated by patients. Future studies are necessary to confirm if this comprehensive approach will result in improved adherence and ultimately in better assistance.

## HIGHLIGHTS

• Development of a novel standard set to be pursued in IIM follow-up, including main outcome concerns of patients and rheumatologists.
• Additional important outcomes only indicated by patients such as controlling pain, fatigue and skin lesions were also included in this standard set.
• The proposed IIM standard set evaluation consists of MOYACT, MMT8, fatigue and pain VAS, HAQ, and level of physical activity.

## Introduction

The Idiopathic Inflammatory Myopathies (IIM) are a group of rare diseases characterized by chronic muscle inflammation and muscle weakness. Associated manifestations, such as skin lesions, arthritis, gastrointestinal and cardiopulmonary involvement, can also be present. The IIM treatment is usually based on glucocorticoids and immunosuppressive drugs that can account for several short and long-term adverse events.^1^^,^^2^

In view of this challenging therapeutic management of autoimmune diseases, Treat to Target (T2T) strategies are becoming the standard approach for several rheumatic disorders. The T2T concept has been widely used in the treatment of chronic disorders, and the achievement of accurate therapeutic targets has led to significant-good long-term prognoses. Adhering to this strategy in clinical practice optimizes the outcomes and facilitates routine follow-up.^3^^,^^4^

However, achieving established outcomes depends on a complex system, which involves the health care providers and, essentially, the patient. In many situations, outcomes are compromised by poor adherence to the proposed treatment. This is particularly relevant in rheumatologic diseases because they are chronic conditions requiring long-term pharmacological treatment with several medications and a high incidence of side effects. In this sense, nonadherence is a recognized problem in the Rheumatology field, reaching up to 48% in IIM patients.^5^^,^^6^

To increase adherence to chronic diseases and to improve the effectiveness of care, patient-centered proposals have been developed.^7^^,^^8^ Introducing the patient into their treatment plan and giving greater value to their concerns related to clinical symptoms and outcomes has been the focus of these proposals. One of the first tools used in this context was the Patient-Reported Outcome Measure (PROM), which consists of self-administered questionnaires filled out by the patients to be used in the decision-making-process by the physician.^8^

In line with the idea of T2T, but also worried with the rising health costs and patients' concerns, the concept of Value-Based Health Care was developed.^9^^,^^10^ Value-based healthcare is a healthcare delivery model in which providers are paid based on health outcomes that matter to patients. But the first step to implementing this model is to develop disease-specifics standard sets of outcome measures. The International Consortium for Health Outcome Measurements (ICHOM) is working on the development of standard sets with the definitions of outcomes for the most prevalent diseases in the world. There is already a well-defined PROM to low back pain, inflammatory arthritis, hip, and knee osteoarthritis, and hand/wrist conditions.^10^

ICHOM is a multidisciplinary team of professionals and researchers that analyzes the perspective of patients, providers, and registries on the disease. Thereby they reach a comprehensive set of potential outcomes and when presenting to patients, prioritize the most important ones. This results in a minimum set of outcomes that are proposed to patients defining feasible, valid, and reliable measures, generating standard sets defined with outcomes and measures.^11^

There are several Clinical Practice Guidelines (CPG) proposed for IIM, but they are sparse and heterogeneous,^1^^,^^2^ and up to now, there is no IIM standard set provided by ICHOM.

Considering that IIM is a condition that can lead to several impairments and a significant reduction in quality of life, clear and measurable targets that address the concerns of patients and physicians are desirable. As for all chronic diseases, adherence is also a problem for IIM, and using PROs as well as defining outcomes together with the patients and a multidisciplinary team may lead to higher adherence levels as well as higher treatment effectiveness.

Therefore, the purposes of this research were to assess the outcome concerns of patients with IIM during the routine follow-up and compare them to the rheumatologists' concerns. Another aim was to gather patients and physicians concerns and develop an IIMs outcome standard set validated by our multidisciplinary team.

## Patients and methods

### Study design

In this observational cross-sectional single-center study, 93 patients were attended regularly in the IIM Clinics of a rheumatology center at a tertiary hospital (Hospital das Clínicas da Faculdade de Medicina da Universidade de São Paulo ‒ HCFMUSP) were consecutively selected from May 2018 to September 2019 and included. In addition, 51 rheumatologists (25 rheumatology fellows and 26 rheumatologists ‒ 4 of them experienced IIM specialists) and one physiotherapist specialist in IIM from our service was also invited in the same period to participate in a standardized questionnaire focusing on IIM outcomes.

Initially, an open questionnaire (Appendix 1) was applied in order to assess unbiased outcome concerns of the two groups (patients and rheumatologists). Subsequently, the top 10 concerns were selected and applied in a multiple-choice questionnaire for both groups (Appendix 2), inquiring the top 3 major concerns to allow further comparisons. Answers of each group were plotted into charts, and frequencies were compared.

The agreement rate was calculated by the sum of the lowest frequency of each concern that appeared in both groups. Concerns were gathered analyzed, and an IIM outcome standard set was developed and validated by patients, rheumatologists, and a physiotherapist, following the first step of the methodology proposed by the ICHOM.^11^ The final standard set was composed of IIM instruments and scores already internationally validated and published.

This study was approved by the local ethics committee (approval n° #13325419.3.0000.0068) and conducted in accordance with the Declaration of Helsinki.

### Study population

Ninety-three consecutive adult IIMs patients were invited to participate in a standardized questionnaire. Patients with dermatomyositis and patients with polymyositis fulfilled the European League Against Rheumatism/American College of Rheumatology (EULAR/ACR 2017) classification criteria,^12^ whereas those with anti-synthetase syndrome met the classification criteria proposed by Connors et al.^13^

### Inclusion criteria

Age more than 18 years, able to read and sign informed consent as well as the questionnaires and PROs.

### Exclusion criteria

Patients with overlapping syndromes and illiteracy were excluded.

### Patients' data

Patients' demographic and clinical/laboratory features were obtained from the study's ongoing standardized electronic chart. We divided these features into 4 domains:

Demographic: Age, gender, self-reported ethnicity (white, yellow, black and mixed), and follow-up time;
Clinical: Myositis subtype (dermatomyositis, polymyositis and anti-synthetase syndrome), ANA positivity (by HEp-2 indirect immunofluorescence), anti-Jo1 positivity (commercially available line blot test kit for Myositis Profile Euroimmun, Lübeck, Germany), serum levels of Creatine Phosphokinase (CPK) (by automated kinetic methods ‒ normal range: 24‒173 IU/L), Manual Muscle Testing (MMT)-8 score,^14^ disease activity rate according to the expert opinion (remission, low activity or high activity), presence of Interstitial Lung Disease (ILD) assessed by high resolution computed tomography, disease damages, and side effects during the last 12 months;
Comorbidities: Obesity, systemic arterial hypertension, diabetes mellitus, neoplasm, and anxiety/depression (all comorbidities were defined by the registered diagnosis in the electronic medical chart);
Medication: Current use of prednisone, intravenous methylprednisolone, intravenous human immunoglobulin, azathioprine, mycophenolate mofetil, methotrexate, cyclophosphamide, cyclosporine, hydroxychloroquine, and rituximab.

### Statistical analysis

Normality was assessed by Kolmogorov-Smirnov test. The quantitative variables were expressed as mean ± Standard Deviation (SD) and categorical results as absolute numbers (%). To compare categorical variables among the groups, either the Chi-Square test or Fisher's exact test were used, when appropriate. All tests were conducted with a significance level of *p* < 0.05.

## Results

### Clinical and demographic features

The mean age of the patients was 48±13 years; 73% were female, 69% were white. The disease duration was 8.0±6.2 years. The demographic features are presented in Table 1.

**Table 1:** Demographic features of patients.

Features	Patients (*n* = 93)
Age, years, mean (SD)	48 ± 13
Women/men, n (%)	68 (73)
Disease duration in years, mean (SD)	8.0 ± 6.2
Disease activity/remission n, (%)	
Remission	52 (56)
Mild activity	26 (28)
Severe activity	15 (16)
Diagnosis n, (%)	
Dermatomyositis	49 (53)
Amyopathic dermatomyositis	14 (15)
Anti-synthetase syndrome	33 (35)
Polymyositis	11 (12)

The IIM subtype distribution was 49 dermatomyositis, 33 anti-synthetase syndromes, and 11 polymyositis. According to IIM experts' impression, at the time of the assessment of the questionnaire, 56% of patients were in remission, 28% were in mild activity, and 16% were in severe disease activity. The mean serum levels of CPK were 339.5±749.3 U/L (median of 118.5, ranging from 26 to 4708), and the mean MMT8 was 78.0±4.0. ANA was detected in 63%, and 22% of the patients were anti-Jo-1 autoantibody-positive.

Analysis of clinical features of IIM revealed 43% presented arthritis, 47% had interstitial lung disease, and 73% of patients had skin lesions. There were 12% of patients with a diagnosis of fibromyalgia, and 19% of patients were using anxiolytics and antidepressants.

IIM treatment was 11% (intravenous methylprednisolone), 10% (intravenous human immunoglobulin), 2% intravenous cyclophosphamide, 14% (rituximab), 11% prednisone (dose ≥20 mg/day). A total of 65% of patients were using one oral immunosuppressive drug, and 20% of patients were using the association of two oral immunosuppressive drugs. The most frequently used drugs were 31% azathioprine, 29% mycophenolate mofetil, 26% methotrexate, followed by 9% cyclosporine, and 11% leflunomide. Continuous analgesic drugs were reported by 35% of patients.

### Patients and rheumatologists' outcome concerns

The top three concerns raised among the patients were a side effect of medication, muscle weakness, and loss of functionality. The top three concerns among rheumatologists were to prevent loss of functionality, ensure the quality of life and achieve disease remission. The list of all domains and answers of patients and physicians is presented in Table 2.

**Table 2:** Comparisons of outcome concerns reported by patients and rheumatologists.

Outcome Concerns	Patients(*n* = 93)	Rheumatologists(*n* = 51)	*p*
Medication side effects, n (%)	47 (51)	9 (18)	0.0001
Muscle weakness, n (%)	46 (49)	20 (39)	0.2946
Functionality, n (%)	33 (35)	36 (71)	0.0001
Muscle pain, n (%)	31 (33)	0	0.0001
Lung manifestations, n (%)	26 (28)	1 (2)	0.0001
Diffuse pain, n (%)	23 (25)	0	0.0001
Skin lesions, n (%)	21 (23)	0	0.0001
Fatigue, n (%)	17 (18)	0	0.0006
Quality of life, n (%)	16 (17)	32 (63)	0.0001
Extra muscular manifestations, n (%)	11 (12)	10 (20)	0.0006
Joint pain, n (%)	8 (9)	0	0.0506
Disease remission, n (%)	0	32 (63)	0.0001
Glucocorticoid dose, n (%)	0	11 (22)	0.0001
Creatine phosphokinase, n (%)	0	2 (4)	0.1238

The agreement rate between patients and rheumatologists was 41%, mostly due to patient functionality, muscle weakness, extra-muscular and pulmonary manifestations, side effects of medications, and quality of life.

### Discrepancies between outcome concerns of patients and rheumatologists

The concerns of patients that rheumatologists did not mention in their top 3 concerns were, respectively, the improvement of muscle pain, widespread pain, skin lesions, and fatigue. The concerns of rheumatologists that patients did not mention were, respectively, the achievement of disease remission, corticosteroid dose, and serum levels of CPK.

### Associations between outcome concerns of patients and clinical features

Some patients' concerns were associated with demographic features, clinical features, comorbidities, and medications (Table 3).

**Table 3:** Associations between patient's outcome concerns and clinical features.

Patients outcome concerns	Demographic features	Clinical features	Comorbidities	Medication
Medication side effects	‒	‒	↑ Anxiety or Depression^a^	‒
Muscle weakness	‒	↑ CPK^a^	↓ Obesity^a^	‒
		↓ ILD^a^		
Functionality	‒	↑ PM^a^	↑ Fibromyalgia^a^	‒
Muscle pain	‒	‒	↑ Anxiety^a^	‒
			↓ Fibromyalgia^a^	
Lung manifestations	‒	↑ ASS^a^	‒	↑ MMF^a^
		↓ Polymyositis^a^		
Diffuse pain	‒	‒	‒	↓ Rituximab^a^
Skin lesions	↓ Mean age^a^	‒	‒	‒
Fatigue	‒	‒	‒	‒
Quality of life	‒	‒	‒	‒
Extra muscular manifestations	‒	‒	‒	‒
Joint pain	‒	‒	‒	‒

ASS, Anti-Synthetase Syndrome; CPK, Creatine Phosphokinase; ILD, Interstitial Lung Disease; MMF, Mycophenolate Mofetil.

a *p* < 0.05.

Patients who were worried about side effects had a higher frequency of anxiety or depression than the other patients (30% vs*.* 11%, respectively; p = 0.023), but they did not have higher rates of side effects in the last 12 months (12% vs. 17%, p = 0.532). Patients who pointed to muscle weakness as concern had higher serum levels of CPK at the time of the research (553.35±1017.03 U/L vs. 125.80±87.90 U/L, p = 0.005), but less ILD diagnosis (35% vs. 60%, p = 0.016). The concern with fatigue had no association with higher serum level of CPK (633.88 ± 548.79 U/L vs. 272.86 ± 1299.16 U/L, p = 0.072).

Of note, the worry with diffuse pain was not associated with fibromyalgia (9% vs. 22%, p = 0.130), but it was associated with the use of rituximab prescription (0% vs. 19%, p = 0.033), whereas worry with functionality was associated with a higher frequency of polymyositis (21% vs. 7%, p = 0.048) and fibromyalgia (21% vs. 7%, p = 0.048). Muscle pain concern was associated with a lower frequency of fibromyalgia (0% vs. 18%, p = 0.010) and a higher frequency of anxiety (13% vs. 2%, p = 0.046).

Patients worried with lung manifestations had a higher frequency of the use of ASS (58% vs. 27%, p = 0.007) and mycophenolate mofetil (46% vs. 22%, p = 0.040). Patients worried with skin lesions were younger than patients that did not point out this concern (41.0 ± 10.7 years vs. 50.1 ± 3.4 years, p = 0.005).

The concerns with fatigue, quality of life, joint pain, and other extra muscular manifestations had no association with demographics, clinical features, and medication used.

### Development of an inflammatory idiopathic myopathy's outcome standard set

**Fig. 1:**
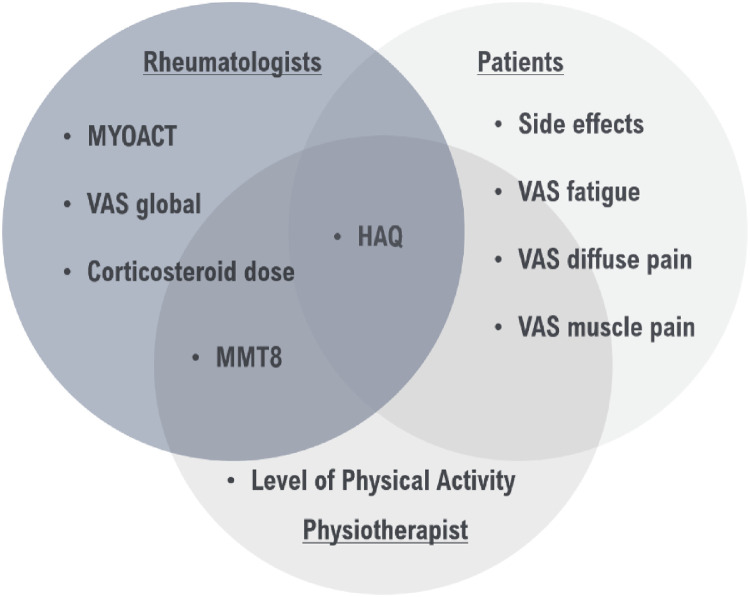
Suggested inflammatory idiopathic myopathies outcome standard set. HAQ, Health Assessment Questionnaire; MMT, Manual Muscle Testing; MYOACT, Myositis Disease Activity Assessment; VAS, Visual Analogue Scale.

Gathering priority choices of patients and rheumatologists allowed the development of an IIM outcome standard set presented in Fig. 1. This standard set was then validated by the IIM specialists (*n* = 4), by a subset of IIM patients (*n* = 30), and by a physiotherapist (*n* = 1). All of them agreed that this could be a useful tool to assess outcomes in IIM, and when patients were asked if there was any other concern not represented by the standard set, there were no additional suggestions.

## Discussion

This study showed that patients and rheumatologists have the same different but complementary perceptions of outcomes in IIM.

Although patients and rheumatologists agreed on several outcome concerns (especially in preventing loss of functionality), some of them, such as fatigue and pain, were not even mentioned by the rheumatologists. Few previous studies analyzed and compared IIM concerns of patients and physicians.^15^^,^^16^ The most relevant was an OMERACT study, with strong patient participation, that reported that fatigue and pain were important patient concerns and considered these items as mandatory in their core set.^15^

An advantage of the present study was that the first assessment of patients was made with an open questionnaire allowing patients to describe items independently of the specialist's knowledge, reducing information bias. After this first assessment, a multiple option survey was designed using unbiased inputs from patients and physicians. Differently, the OMERACT study used the Delphi methodology to develop a PROM core set.^15^ The most important difference between the two sets is that the OMERACT intended to develop a patient-reported outcome instrument, and the current set was developed as a guide to standardize the IIM patient follow-up gathering both points of view: the patients (using the PROM) and specialists (using the validated instruments to assess disease activity) as suggested by the ICHOM.^11^

In addition, the currently developed IIM Outcome Standard Set included the assessment of disease activity (using MYOACT instrument, global VAS, MMT8 and prednisone dose), quality of life (using HAQ questionnaire), side effects, pain (diffuse and muscle VAS), fatigue's VAS and physical activity. The OMERACT PROMs mandatory domains were muscle symptoms, adverse events, physical activity, pain, and fatigue.^15^

Usually, Clinical Practice Guidelines (CPGs) are responsible for the standardization of care, and they are developed to assist decisions of practitioners and patients about appropriate healthcare for specific clinical circumstances.^1^ A recent review stated that none of the existing IIM CPGs takes into consideration patient's preference or PROMs, and in general, they are neither validated together with a multidisciplinary team, nor do they discuss comorbidities prevention.^1^ This currently proposed Standard Set covers most of these frailties.

The present study is the first to evaluate if there is an association between a demographic, clinical, treatment, or comorbidity parameter with IIM patient's outcome concerns. In this regard, 20% of the patients evaluated herein had depression or anxiety diagnosis, a figure lower than the 44% (18/41) reported in a previous study in patients with DM.^17^ The analysis of the impact of the above parameters revealed that worry with fatigue or pain was not associated with a higher frequency of fibromyalgia, depression, or other comorbidities in IIM patients. Conversely, concern with side effects was associated with a higher frequency of anxiety or depression in spite of the fact that this group of patients did not have a higher frequency of previous side effects in the last 12 months.

In IIM, adverse events and treatment-related comorbidities (i.e., infections, osteoporosis, cardiovascular events, and high-risk pregnancy) are major causes of morbidity and mortality in patients with IIM.^1^ Thereby, it is understandable that patients chose as top 1 concern the side effects of drugs. This is also a worry of physicians, particularly related to prednisone dose.

This divergence in opinion between professionals and patients, such as the focus on remission and CK levels from rheumatologists and the focus on current symptoms from patients, may show that the dialogue and perceptions of improvement between the two groups can be different, but methods to improve the doctor-patient relationship can help to approximate and reduce the disparity between these perceptions.

As a limitation of the present study, the research was single-centered with a small representation of other professional categories, and it would be interesting to validate this proposed IIM standard set to other centers with a multidisciplinary team.

In conclusion, even though muscle weakness, functionality, quality of life, and disease remission emerged as major outcome concerns of physicians and patients, there are additional patients concerns that should be assessed routinely during IIM treatment and follow-up. Patients consider that controlling pain, fatigue, and skin lesions are important outcomes to be pursued in IIM, and these concerns were included in the present study's standard set. Therefore, rheumatologists should be aware of these concerns to provide better assistance and ensure treatment adherence.

## Authors' contributions

Bertoglio IM, Abrahao GF, Bonfa E, Lopes MRU, conceptualization, data curation, writing - original draft, review & editing. Souza FHC, Miossi R, Moraes PC, Shijo SK, writing - review & editing.

## Conflicts of interest

The authors declare no conflicts of interest.

## Appendix Supplementary materials

Image, application 1
