# Supplementary material for: Gathering patients and rheumatologists' perceptions to improve outcomes in idiopathic inflammatory myopathies
Source: Clinics (Sao Paulo). 2022 Apr 11;77:100031. doi: 10.1016/j.clinsp.2022.100031 (PMC9020087; doi:10.1016/j.clinsp.2022.100031)
Supplement: Supplementary file 1 [file mmc1.pdf]

CLINICS-2021-3405_Supplementary Material

Appendix 1 Open questionnaire.

Dear patient,<br>We are conducting research to better understand your concerns regarding your disease. Although currently there is no cure for IIM, it can be controlled to ensure a good quality of life. In order to understand your concerns about your disease and what your expectations of outcome are, please answer the following questions:		
1) Regarding IIM symptoms, what are your concerns?		
2) What are the complications of your disease that you fear the most?		
3) Does your illness bring you any limitations?	( ) No	( ) Yes
What are your concerns on this subject?		
4) Are you concerned with side effects? Which ones?		
5) Is there anything else, any outcome that you think that is important and your doctor doesn't usually pay attention?		
( ) No	( ) Yes.	If Yes, what?

Appendix 2 Multiple-choice questionnaire.

Dear Rheumatologist/Patient,<br>Please check the 3 most important outcome concerns for monitoring and treating myopathies:	
	**Top 3 Outcome Concerns**
	Medication side effects
	Muscle weakness
	Functionality
	Muscle pain
	Lung manifestations
	Diffuse pain
	Skin lesions
	Fatigue
	Quality of life
	Extra muscular manifestations
	Joint pain
	Disease remission
	Corticosteroid dose
	Laboratory test (creatine phosphokinase)
